# Congenital aniridia: European COST action ANIRIDIA‐NET guidelines for diagnosis, management and care

**DOI:** 10.1111/aos.17587

**Published:** 2025-09-02

**Authors:** Davide Romano, Vito Romano, Alejandra Daruich, Giulio Ferrari, Matthieu Robert, Francesco Semeraro, Neil Lagali, Dominique Bremond‐Gignac, the ANIRIDIA‐NET Clinical Guidelines Working Group, Miriam Barbany, Claus Cursiefen, Stefano Ferrari, Francisco Figueiredo, Christina Grupcheva, Jesper Hjortdal, Elke Kreps, Erlend Landsend, Laura Mauring, Hilde Pedersen, Nora Szentmáry, Bogumil Wowra, the Aniridia‐Europa and Geniris Working Group, Sophie Valleix, Juan Álvarez de Toledo, James Lauderdale, Tor Paaske Utheim, Paolo Rama

**Affiliations:** ^1^ St. Paul's Eye Unit, Department of Corneal Diseases Royal Liverpool University Hospital Liverpool UK; ^2^ Department of Eye and Vision Sciences University of Liverpool Liverpool UK; ^3^ Eye Clinic, ASST Spedali Civili di Brescia, Department of Medical and Surgical Specialties, Radiological Sciences, and Public Health University of Brescia Brescia Italy; ^4^ Ophthalmology Department Necker‐Enfants Malades University Hospital, AP‐HP, Paris Cité University Paris France; ^5^ INSERM, UMRS1138, Team 17, From Physiopathology of Ocular Diseases to Clinical Development Sorbonne Paris Cité University, Centre de Recherche des Cordeliers Paris France; ^6^ Division of Neuroscience, Cornea and Ocular Surface Disease Unit, Eye Repair Lab IRCCS San Raffaele Scientific Institute Milan Italy; ^7^ Department of Biomedical and Clinical Sciences Linköping University Linköping Sweden

**Keywords:** aniridia, aniridia glaucoma, aniridia guidelines, aniridia management, aniridia outcome, aniridia surgery, *PAX6 gene*

## Abstract

Congenital aniridia is a rare ocular disorder affecting the majority of eye structures and can be associated with systemic manifestations. The main visible phenotypic characteristic is the partial or complete absence of the iris; however, foveal hypoplasia is a more frequent and reliable clinical sign. Other ocular comorbidities are associated with the disease, such as cataract, keratopathy and optic nerve hypoplasia. Mutation in the *PAX6* gene is the most common cause of congenital aniridia, but other causative mutations exist. The main ocular symptoms experienced by those with congenital aniridia are photophobia, glare, low visual acuity, dryness/irritation of the ocular surface and nystagmus. Management and follow‐up of patients with congenital aniridia can be challenging due to the lack of effective therapeutic options and the complexity of ocular manifestations and outcomes. These guidelines for the diagnosis, management and care of congenital aniridia have been developed at the European level, thanks to ANIRIDIA‐NET, based on a review of the scientific literature on clinical and molecular characterization, therapeutic options as well as non‐medical approaches.

## 1 INTRODUCTION

Congenital aniridia is a rare panocular disorder with a global incidence of 1:48.000 to 1:96.000 and is classically characterized by the partial or complete absence of the iris (Landsend et al., 2021; Daruich, Duncan, et al., 2022). Congenital aniridia, however, is a complex eye disease, encompassing more than just the iris or the eye, and necessitates a multidisciplinary approach for proper management. Iris and foveal hypoplasia are the most prevalent features, followed by a progressive aniridia‐associated keratopathy (AAK), nystagmus, congenital and early onset cataract, glaucoma and optic nerve hypoplasia (Daruich, Duncan, et al., 2022; Lagali et al., 2020b). The severity of signs can vary from one individual to another, even within the same family. Prognosis for vision is generally poor in adulthood, with most patients being severely visually impaired or legally blind.

Genetically, congenital aniridia can present as part of a known syndrome, presenting with extra‐ocular manifestations of a pronounced nature that require clinician management.

In other cases, congenital aniridia may not be associated with an established syndrome but may nevertheless be characterized by predominantly ocular, but also extra‐ocular abnormalities.

Congenital aniridia is caused most frequently by a heterozygous mutation in the *PAX6* gene on chromosome 11 (11p13) (Daruich, Robert, et al., 2022), accounting for roughly around 90% of the genetic anomalies reported in various clinical studies.

One‐third of cases result from spontaneous de novo sporadic mutations, with the remaining two‐thirds being inherited in an autosomal‐dominant pattern (Netland et al., 2011). The *PAX6* gene plays a key role in the development of the eye and its surrounding extra‐ocular structures (Lim et al., 2017), and therefore, mutations in *PAX6* can lead to a variety of ocular manifestations. Because *PAX6* is also expressed in the developing brain and pancreas, in addition to the ocular manifestations, cerebral and central nervous system manifestations as well as impaired glucose regulation may be prevalent among those with *PAX6* mutations (Grant et al., 2021; Peter et al., 2013; Tian et al., 2021).

Other aniridia‐causing mutations not involving *PAX6* mutation are less frequent and involve the genes *CYP1B*, FOXC*1, FOXD3, PITX2* and *TRIM44*—a mutation in any of these genes can lead to different aniridia ocular phenotypes, with each mutated gene also having its own pattern of extra‐ocular involvement (Samant et al., 2016).

Contiguous deletion of both *PAX6* and *WT1* genes leads to Wilms' tumour as part of WAGR syndrome (Wilms' tumour, aniridia, genitourinary anomalies and intellectual deficit) (Samant et al., 2016). Gillespie syndrome, causing cerebellar ataxia and oligophrenia, associated with *ITPR1* gene anomalies, can also rarely be associated with congenital aniridia (Gerber et al., 2016; Samant et al., 2016).

In view of the wide phenotypic heterogeneity and complexity of congenital aniridia, our aim was to review the available scientific literature and knowledge, to provide a harmonized guideline for diagnosis, management and care of patients with congenital aniridia.

## 2 METHODS

We reviewed recommendations and guidelines for aniridia, including European nationals' guidelines updated in the last 5 years (January 2020–December 2024), which included French national protocol for diagnosis and care of congenital aniridia and Sweden national guidelines (Bremond‐Gignac et al., 2022; Lagali et al., 2023).

The research was conducted by the PICO (Population, Intervention, Comparison, Outcome) method (Cooke et al., 2012).

A single investigator (DR) used the MEDLINE database (via PubMed) to search for and identify articles for inclusion in this review.

Key words used were ‘congenital aniridia’ AND ‘genotype’, ‘management’, ‘complications’, ‘outcome’, ‘cornea’, ‘glaucoma’, ‘cataract’, ‘keratopathy’, ‘iris devices’, ‘epidemiology’, ‘treatment’, ‘follow‐up’.

The research was limited to 16 years (January 2009–May 2025) in light of the richness and abundance of bibliographic material in the field in recent years and newer knowledge and methods updating earlier studies.

Exceptions were made for the outcomes of ‘prophylactic goniotomy’ and ‘cyclodestructive laser treatments’, as these therapeutic approaches are historically reported in literature with no updated studies.

## 3 DIAGNOSIS

The initial diagnosis of aniridia is based on clinical signs and is often suggested in the first few months of life, or occasionally later in childhood when characteristic features become apparent (Landsend et al., 2021).

During the first months of a child's life, parents may seek a paediatric consultation due to various symptoms of suspected visual impairment, such as nystagmus, photophobia, dark colour of eyes (especially in families with light colour of irises), wandering gaze, buphthalmos (sign of congenital glaucoma), corneal opacity, leukocoria (sign of congenital cataract) or microphthalmia (Rahi & Cable, 2003).

In case of positive family history for aniridia, paediatric and ophthalmology consultations are highly recommended (Bremond‐Gignac et al., 2022).

Upon suspicion of aniridia, the patient should be referred to an ophthalmologist who confirms the diagnosis through a comprehensive assessment of ocular abnormalities. Ideally, the ophthalmologist will refer the patient to a specialized reference centre to ensure proper management of the disease (Bremond‐Gignac et al., 2022). Multidisciplinary collaboration is necessary for the diagnosis, initial assessment and overall management of the patient, led by the ophthalmologist at the reference centre. The involvement of a range of professionals depends on the associated manifestations, whether ocular or extra‐ocular, and ideally, on the genotype of the congenital aniridia (Bremond‐Gignac et al., 2022).

Genetic testing and counselling are highly recommended and should always be requested in cases of family history of aniridia or clinical suspicion of aniridia, as genetic results help to determine the risk of Wilms' tumour or other known syndromes, for improved subsequent management and care, while counselling helps to inform on the risk of inheritance (Tam et al., 2023; Wawrocka & Krawczynski, 2018).

If the diagnosis occurs during childhood, a full paediatric assessment is performed to detect any systemic abnormalities and exclude the possibility of WAGR syndrome (Daruich, Robert, et al., 2022; Landsend et al., 2018).

A classic regular cytogenetic analysis using a *PAX6* probe may be carried out to look for a deletion, with regular renal ultrasounds performed while awaiting results to check for Wilms' tumour (Bălănescu et al., 2022; Blanco‐Kelly et al., 2021). Subsequently, a clinical medical genetic consultation is advisable to identify mutated genes and/or to determine if an associated syndrome (e.g. WAGR or Gillespie syndrome) is present (Obst et al., 2025).

In the presence of suspected developmental delay, a neurological examination with magnetic resonance imaging (MRI) should be requested to exclude the presence of cerebellar abnormalities, suggestive of Gillespie syndrome (Hall et al., 2019).

### 3.1 First ophthalmological evaluation and follow‐up assessment

The initial ophthalmological work‐up in case of referral for suspected congenital aniridia is made by simple, non‐invasive investigations, aiming to collect a wide range of information, and, in case of predominantly ocular abnormalities, is to perform the differential diagnosis with developmental abnormalities (dysgenesis) of the anterior segment (Kuang et al., 2023; Vanathi et al., 2022).

These include: Axenfeld–Rieger anomaly/syndrome; Peters' anomaly/syndrome; congenital glaucoma; iris coloboma; colobomatous microphthalmia; correctopia; oculocutaneous albinism (OCA) and ‘pure’ ocular albinism; congenital corneal opacities; iridocorneal endothelial syndrome (ICE); anterior segment dysgenesis, anterior chamber cleavage syndrome (Idrees et al., 2006).

Initially, it is recommendable to collect a complete anamnesis: history of eye disease (glaucoma, cataract, general (WAGR, etc.)), medical treatments (topical lubricants, autologous serum, glaucoma treatments, other drugs), surgical history and family history and pedigree of congenital anomalies of eye development.

Subsequently, a complete eye examination must be performed, considering that congenital aniridia is a panocular disorder, affecting to various degrees the eyelids, meibomian glands, tear film, ocular surface including cornea, iris, iridocorneal angle, lens, ocular alignment, fovea and optic nerve (Alafaleq et al., 2023; Landsend et al., 2019, 2021).

The phenotype can vary both among and within families (Kit et al., 2021). Nonetheless, in affected patients, minimal variation between the two eyes is noted, although asymmetry in the keratopathy can be present in about 30% of cases (Lagali et al., 2018). Patients commonly experience nystagmus, reduced visual acuity (generally ranging from 20/100 to 20/1000 in adulthood) and foveal hypoplasia (Alafaleq et al., 2023; Daruich, Robert, et al., 2022).

Additionally, although less common, mild forms of aniridia exist, with fine anomalies of the iris, no visual impairment and normal foveal structure (Hingorani et al., 2009). Usually, severe forms present with corneal involvement, glaucoma, cataract, strabismus, hypoplasia or coloboma of the optic nerve and microphthalmia (Dentel et al., 2024; Landsend et al., 2021).

In early stages, foveal hypoplasia is the main cause of reduction of visual acuity; however, in later stages, worsening of visual acuity is related to progression of cataract, glaucoma and corneal opacification (keratopathy), often leading to legal blindness (Daruich, Duncan, et al., 2022).

In most children, early signs of aniridia appear at birth or in the first few months of life, including iris or pupil abnormalities with or without accompanying nystagmus. Congenital glaucoma and/or megalocornea are rare manifestations of *PAX6‐*related congenital aniridia (Lipsky & Salim, 2011). Congenital corneal opacity is also a rare manifestation of aniridia (Karadag et al., 2020; Lee et al., 2018).

When congenital aniridia is suspected in a child, a complete ophthalmological examination under general anaesthesia is recommended at first examination.

The first step of clinical examination includes inspection of the face to look for ptosis (with rating of the severity of any palpebral ptosis present), near and distance visual acuity unaided, best‐corrected visual acuity and cycloplegic refraction. Additionally, orthoptic assessment is advisable to assess the nature of nystagmus and possible presence of strabismus or heterophoria (Alafaleq et al., 2023).

The second step involves slit‐lamp examination, with photography of the anterior segment for monitoring, especially the cornea and the limbus, in view of the aniridia‐associated keratopathy (AAK). At slit lamp, the following structures should be assessed: cornea, 360° of the limbus, iris and lens.

Ocular surface including cornea and limbus: dry eye has a prevalence of 56%–96% in congenital aniridia, while aniridia‐associated keratopathy, defined as opacification and neovascularization of the cornea indicating limbal stem cell deficiency (LSCD), has a prevalence between 78% and 96%. A minimal keratopathy, with neurotrophic and inflammatory changes in the cornea, has been reported to be present in 100% of cases (Lagali et al., 2013; Lee et al., 2018). In children, LSCD is usually more frequently observed in its early stages, with vessel invasion starting at the limbal region of the cornea (Landsend et al., 2021).

AAK subsequently leads to impairment of vision through progressive opacification of the cornea concomitant with neovascularization, pain and photophobia (Lagali et al., 2020a; Latta et al., 2021). AAK is associated with an insufficiency or functional deficiency of limbal epithelial stem cells resulting in progressive invasion of the cornea by conjunctival epithelium and neovascularization (Bremond‐Gignac et al., 2018; Li et al., 2025; Nastaranpour et al., 2025; Stachon, Fecher‐Trost, et al., 2024; Stachon, Latta, et al., 2024). Clinical signs of AAK include a gradual breakdown of the palisades of Vogt, irregular opaque corneal epithelium, increased permeability of the cornea to fluorescein, epithelial defects, superficial corneal neovascularization and stromal fibrosis (Lagali et al., 2013).

Sub‐basal corneal nerve plexus, keratocyte density and endothelium are also affected in the case of AAK.

Significantly reduced corneal nerve fibre density, length and branching, along with thicker nerve fibres, and lower keratocyte density has been reported (Csorba et al., 2024), as well as corneal endothelial cells with reduced diameter, altered spatial arrangement and the presence of hyperreflective deposit, correlated with the severity of AAK (Csidey et al., 2025).

**FIGURE 1:**
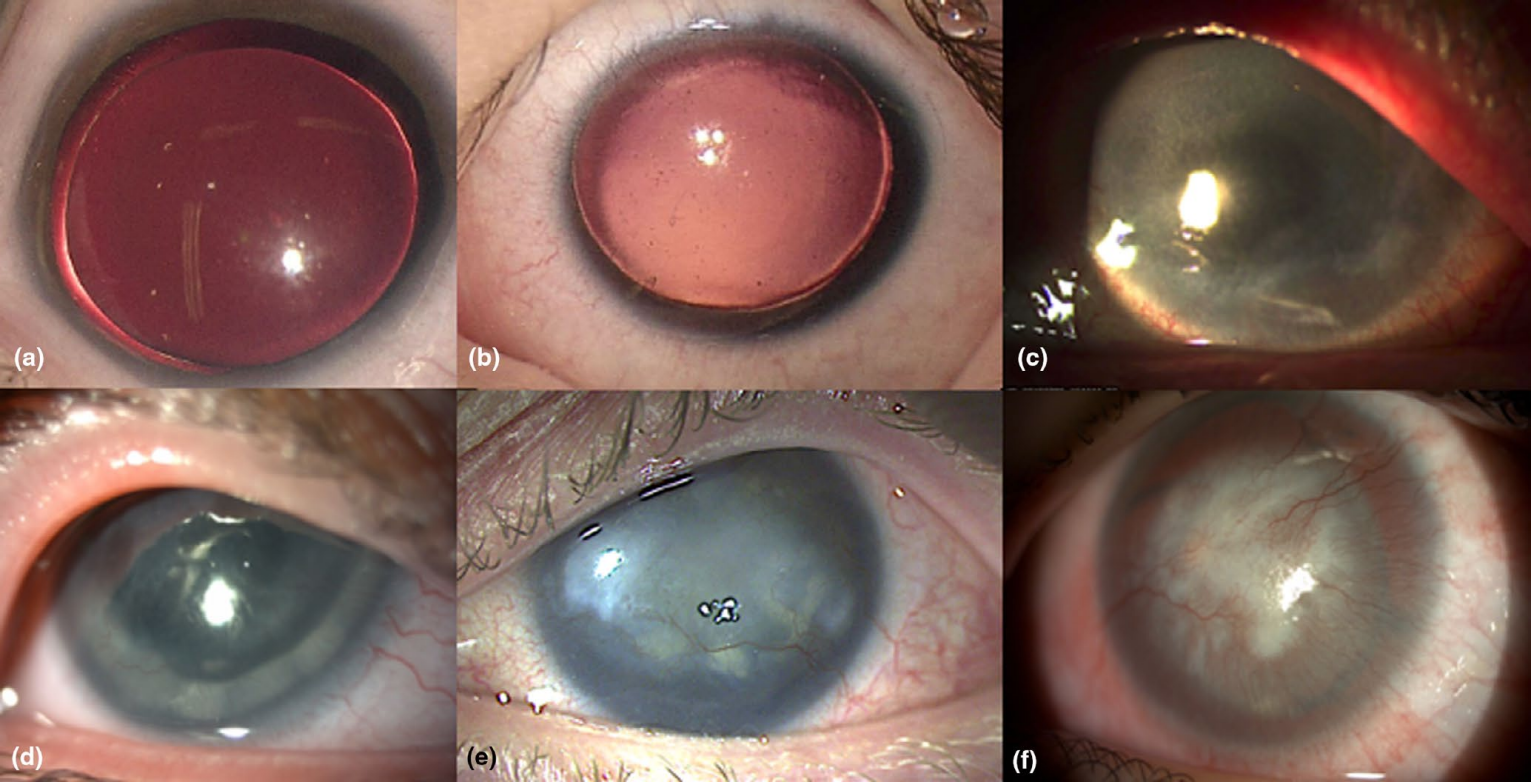
Representative images of aniridia‐associated keratopathy (AAK) grading: (a) Grade 0—clear cornea; (b) Grade 1—vessels and conjunctival tissue cross the limbal border; (c) Grade 2—vessels and conjunctival tissue invade peripheral and mid‐peripheral cornea, without involving the central 2–3 mm of cornea; (d) Grade 3—advancing opacity/vascularization toward the visual axis; (e) Grade 4A—dense central involvement; (f) Grade 4B—complete central opacity with severe vascularization.

For the grading of AAK, a four‐point scale is useful (Table 1) (Lagali et al., 2020a). In Grade 0, the limbal border is intact, with no conjunctival tissue or vessels crossing the limbus. In Grade 1, vessels and conjunctival tissue cross the limbal border within approximately 1 mm from the limbus. The invasion can be localized to one region of the limbus, with other areas of the limbus remaining intact. In Grade 2, conjunctival tissue with vessels invades the peripheral and mid‐peripheral cornea, without affecting the central 2–3 mm of cornea. In Grade 3, conjunctival tissue also invades the central cornea (spared in Grade 2), subsequently affecting the central visual axis. Typically, a translucent corneal pannus covers the entire corneal surface. In Grade 4, the pannus becomes opaque, vascularized and thick. In Grades 0–2, central corneal transparency is not impacted, while Grades 3 and 4 affect the central vision (Figure 1).

**TABLE 1:** Grading scheme for aniridia‐associated keratopathy.

AAK grade	Limbal border	Central visual axis involved	Findings
0	Not affected	Not involved	No abnormalities
1	Affected <1 mm	Not involved	Vessels and conjunctival tissue cross the limbal border
2	Affected >1 mm	Not involved	Vessels and conjunctival tissue invade peripheral and mid‐peripheral cornea, without interest the central 2–3 mm of cornea
3	Affected >1 mm	Involved	Vessels and conjunctival tissue invade also the central cornea
4	Affected >1 mm	Involved	Same of Grade 3 and presence of opaque, vascularized and thick central corneal pannus

Another corneal finding is increased central corneal thickness (Lagali et al., 2013, 2020b). Central corneal thickness of typically 600 μm was reported in early‐stage AAK, increasing to 1 mm in later stages, where a normal or above‐normal endothelial cell density was reported, and it was hypothesized that an inability to complete the normal developmental thinning cycle of the cornea in prenatal stages can lead to the observed corneal thickening in aniridia (Lagali et al., 2020a).

Iris and pupil: The most noticeable defect is partial or complete absence of the iris. In some cases, the rudimentary iris root may be visible via gonioscopy, or with devices such as anterior segment optical coherence tomography (AS‐OCT) and high‐frequency ultrasound biomicroscopy (UBM) (Alexander et al., 2021). In less severe cases, the size of the pupil may be normal, but the typical surface features of the iris may be lost or there may be iris transillumination (Hingorani et al., 2009).

Additional iris anomalies may include partial iris defects, resembling a coloboma or pupils that are eccentric or misshapen, and iris ectropion.

Lens: cataract is common in congenital aniridia, with prevalence reported of up to 90% of patients (Edén et al., 2014; Park et al., 2010). It presents as congenital lens opacities in the child, while in the adult, it may progress to a fully opaque cataract (Landsend et al., 2021). Adult patients can present with cartwheel cataract, a specific feature in *PAX6*‐related aniridia (Daruich, Robert, et al., 2022; Edén et al., 2014). Other lens abnormalities include lens dislocation, lens coloboma, microspherophakia and posterior lenticonus (Duncan et al., 2024; Landsend et al., 2021).

Fundus examination: The most common findings are foveal hypoplasia (prevalence 79%–92%, manifested at ophthalmoscopy as reduction of foveal reflex, macular hypopigmentation and presence of retinal vessels in the central avascular zone of the fovea), light fundus colour (prevalence 86%) and optic nerve hypoplasia (prevalence 11%–29%) (Landsend et al., 2021).

The measurement of intraocular pressure (IOP). Glaucoma is among the most common ocular comorbidities in patients with congenital aniridia, with a prevalence of over 50% (Balekudaru et al., 2017; Landsend et al., 2021). Congenital glaucoma, however, is not so common in *PAX6*‐related aniridia. Usually, glaucoma is not present at birth, so there is a lack of common signs of congenital glaucoma such as Haabs striae and/or buphthalmos, while glaucoma arises more frequently during childhood or adolescence (Badawi et al., 2019; Landsend et al., 2021). The prevalence of glaucoma is around 15% in the age group below 10 years, with the mean age at diagnosis around 15 years; however, this may be an underestimation (Landsend et al., 2021).

The pathogenesis of glaucoma in cases of congenital aniridia is often due to dysgenesis of the trabecular meshwork or to its obstruction by stromal iris roots which progressively migrate toward the iridocorneal angle forming synechiae (Grant & Walton, 1974; Walton, 1986). A further cause of glaucoma can also be the absence of Schlemm's canal (Landsend et al., 2021).

Regular monitoring of the iridocorneal angle with gonioscopy and measurement of IOP are required, at intervals depending on the age, but usually every 6 months, and measurement readings should be corrected by corneal pachymetry, due to increased corneal thickness in congenital aniridia that also varies with the grade of AAK, as mentioned above (Lagali et al., 2020a).

Additional investigations with ophthalmic equipment may be beneficial; however, it should be considered that examinations such as optical coherence tomography (OCT), corneal topography/tomography and in vivo confocal microscopy (IVCM) may be difficult to perform or give inconsistent results in view of nystagmus and/or AAK. Handheld devices may be useful, especially in children.

High‐frequency UBM is helpful in cases of advanced corneal opacification to demonstrate the partial or total absence of iris (Nischal, 2002). Anterior segment OCT (AS‐OCT) provides details of the anatomy of anterior segment structures such as iridocorneal angle, cornea, iris and lens; macular OCT, however, can be used to better detect and grade foveal hypoplasia compared to fundus ophthalmoscopy (Dada et al., 2007; Daruich, Robert, et al., 2022; Dentel et al., 2023).

Despite glaucoma being one of the most common ocular comorbidities, visual field examination may not be reliable not only due to nystagmus and AAK, but also because of visual acuity, which is often significantly impaired in congenital aniridia, and, in the case of children, examining visual fields may be difficult due to the level of patient cooperation required.

### 3.2 Follow‐up

To determine the progression of the disease at follow‐up visits, the following examinations are recommended: visual acuity measurement, ocular motility and alignment exploration, assessment for corneal disease, cataract and glaucoma. These are the main factors that may lead to the development of severe visual impairment and blindness in congenital aniridia.

For children under the age of 8, follow‐up appointments should be scheduled every 6 months, especially to measure refractive errors, amblyopia, ocular alignment and monitoring of intraocular pressure. Thereafter, follow‐up appointments should be annual.

The steps of diagnosis, clinical evaluation and follow‐up are summarized in Table 2.

**TABLE 2:** Summary of diagnosis, clinical evaluation and follow‐up.

Step	Description	Key outcomes/prevalence
Initial clinical signs	Nystagmus, photophobia, dark iris colour, wandering gaze, buphthalmos, corneal opacity, leukocoria, microphthalmia	Signs present at birth or early infancy
Family history	If positive, early paediatric and ophthalmology consultations are highly recommended	2/3 autosomal dominant, 1/3 de novo mutations
Genetic testing	Detects *PAX6* mutations (90% cases), identifies risk of Wilms' tumour or Gillespie syndrome	Early testing guides prognosis and screening
Systemic assessment	Paediatric exam, renal ultrasound, neurological exam/MRI if developmental delay	Wilms' tumour risk with *PAX6* and *WT1* deletions; MRI detects cerebellar anomalies for Gillespie syndrome
Ophthalmological work‐up	Full history, eyelids, tear film, cornea, iris, angle, lens, fovea, optic nerve	Foveal hypoplasia 79%–92%, optic nerve hypoplasia 11%–29%
Functional testing	Visual acuity, cycloplegic refraction, orthoptic assessment	Mean adult VA 20/100–20/1000
Imaging	AS‐OCT/UBM for anterior structures, fundus exam, macular OCT	Macular OCT better for foveal hypoplasia grading
IOP monitoring	Gonioscopy and IOP every 6 months, corrected for corneal thickness	Glaucoma prevalence >50% overall; mean diagnosis age ≈15 years
Follow‐up	<8 years: every 6 months; ≥8 years: annually	Early detection prevents amblyopia and late glaucoma

Abbreviations: AS‐OCT, anterior segment optical coherence tomography; IOP, intraocular pressure; MRI, magnetic resonance imaging; OCT, optical coherence tomography; UBM, ultrasound biomicroscopy; VA, visual acuity.

## 4 MEDICAL AND SURGICAL TREATMENT

Currently, there is only palliative care (symptom relief) and no curative treatment for congenital aniridia.

Along with treatments specific for the comorbidities, which will be reviewed in dedicated paragraphs below, the first preventive measure in aniridia is regular examination and correction of refractive errors. Table 3 summarizes the medical and surgical treatment findings and outcomes.

**TABLE 3:** Medical and surgical management of congenital aniridia.

Problem	Medical management	Surgical/other options	Notes
Refractive errors	Glasses (tinted/photochromatic)	—	Improves vision and photophobia; early correction reduces amblyopia risk
Amblyopia	Occlusion therapy or Ryser penalization	Low vision aids (magnifiers, telescopes, video magnifiers)	Best results in early childhood; limited effect in severe foveal hypoplasia
Photophobia	Tinted/photochromic glasses; avoid contact lenses	PID	PID helps in photophobia reduction; risks: glaucoma progression, glaucoma onset, AAK progression, endothelial cell loss
Dry eye/early LSCD	Lubricants, cyclosporin 0.1%, autologous/allogenic serum drops	Scleral lenses (non‐contact)	Maintains ocular surface and delays LSCD progression
Advanced LSCD/AAK	Supportive ocular surface therapy	LSCT (KLAL, lr‐CLAL) Corneal transplant ± LSCT Boston KPro	Visual gain modest; high risk of rejection, glaucoma worsening, extrusion; immunosuppression needed
Glaucoma (early)	Beta‐blockers, prostaglandins, CA inhibitors; avoid brimonidine <8–12 years (causes lethargy, sleepiness)	—	Requires gradual introduction, Medical therapy delays surgery; prostaglandins effective ≥9 years, beta‐blockers first line <9 years preservative‐free preferred
Glaucoma (surgical)	—	Baerveldt implant Ahmed implant trabeculectomy goniotomy	Drainage devices have most favourable outcomes but limited evidence
Glaucoma (end‐stage)	—	Cyclocryotherapy Diode CPC	High complication risk; last‐resort therapy
Cataract	—	Cataract surgery with capsular staining, small capsulorhexis	Delay surgery until severe opacity Risks of glaucoma onset/worsening, LSCD acceleration and endothelial decompensation

Abbreviations: AAK, aniridia‐associated keratopathy; Diode CPD, diode laser cyclophotocoagulation; KLAL, keratolimbal allograft; lr‐CLAL, transplantation and living‐related conjunctival‐limbal allograft transplantation; LSCD, limbal stem cell deficiency; LSCT, limbal stem cell transplant; PID, prosthetic iris device.

Assessment of visual acuity may be difficult, in view of nystagmus or the age of the child, the extent of the iris stump, the presence of foveal hypoplasia and optic nerve involvement.

Refractive abnormalities should be corrected using corrective eyeglasses, either tinted or photochromatic to reduce light sensitivity. Use of soft contact lenses is strongly discouraged because of the local hypoxia created at the ocular surface that can trigger or accelerate the LSCD (Rossen et al., 2016).

In case of amblyopia, occlusion therapy or Ryser optical penalization must be discussed considering the nystagmus, and optical low vision aids are beneficial in case of severe visual impairment (de Zárate & Tejedor, 2007; Perrault et al., 2023).

Management options for the ocular comorbidities in aniridia are the following.

### 4.1 Photophobia

The partial or total absence of iris results in a defective pupillary diaphragm, with subsequent increased sensitivity to light, resulting in reduced vision, but also discomfort, pain and headaches.

Management of photophobia, which usually worsens with aging, can be aided with tinted or photochromic lenses. Any type of contact lens (coloured, tinted or prosthetic) should be discouraged in view of the poor condition of the ocular surface, despite their ability to reduce light sensitivity and provide an improved cosmetic appearance. Different from contact lenses, non‐contact scleral lenses can be beneficial.

Intraocular surgery, with use of prosthetic iris devices (PID) in the form of iris‐lens diaphragm (ILD), capsular tension ring‐based prosthetic iris device (CTR‐based PID) and customized artificial iris (AI), represents further options (Romano et al., 2023).

Considering the manufacturers and up‐to‐date market availability of the PIDs, in the case of ILD, there is the foldable acrylic ILD with CE mark, but not FDA approved, produced by Reper (Reper‐NN Ltd., Nizhniy Novgorod, Russia) and distributed in Europe by Ophtec (Ophtec BV, Groningen, Netherlands), and in the case of the artificial iris, the CustomFlex artificial iris (HumanOptics AG, Erlangen, Germany) is available, which has been sold as a custom‐made device since 2002 until it received CE mark in 2011 and FDA approval in 2018.

The artificial iris manufactured by BrightOcular (Stellar Devices, New York, USA) did not receive FDA approval or the CE mark, and it is associated in healthy eyes with serious ocular complications, including uveitis, glaucoma and corneal decompensation, with possible blinding consequences (Ghaffari et al., 2021; Koaik et al., 2018; Mansour et al., 2016; Mathew et al., 2019; Mednick et al., 2018; Shalash & el Bahrawy, 2020; Varna‐Tigka et al., 2020).

PIDs have been shown to reduce photophobia, with a reduction of symptoms by 96% in the case of the CustomFlex artificial iris and 90% in the case of ILD, and are also associated with an increase in visual acuity in 62.5% of patients, with the degree of acuity improvement generally limited to 2–3 logMAR lines of vision (Romano et al., 2023).

Complications after PIDs include glaucoma progression in patients with pre‐existing glaucoma (58.9%), secondary glaucoma (27.6%), progression of AAK (27%), prosthesis decentration (10.6%) and endothelial cell loss (9% and reported only with ILD) (Romano et al., 2023).

Evaluating the efficacy of PIDs on the improvement of visual acuity, however, should be avoided, as any visual gain is most likely related to the concomitant cataract surgery at the time of PID implantation, rather than to the iris implant itself (Romano et al., 2023).

PID implantation should be very carefully considered, and not be proposed as first‐line surgical treatment, as in most cases, the risk outweighs the potential benefit. Detailed counselling with patients is therefore of utmost importance.

### 4.2 Ocular surface

#### 4.2.1 Dry eye

Use of preservative‐free lubricants is recommended as soon as the first signs of AAK are detected at ophthalmological evaluation, commonly in infants and young children.

In case of more advanced dry eye, additional measures are the use of Cyclosporin 0.1% or allogenic/autologous serum‐eye drops (Deshmukh et al., 2021; Farah et al., 2021; Jones et al., 2017; López‐García et al., 2008; Yazdanpanah et al., 2020).

Scleral lenses may be beneficial, as these are not in direct contact with the cornea or limbus and provide protection for the cornea while keeping the eye lubricated; however, they can be difficult to adjust to the patient's eye (Yazdanpanah et al., 2020). Given the importance of the tear film for the maintenance of the ocular surface and for avoidance of corneal complications, focus must be given on stabilizing and supporting a healthy tear film in aniridia patients generally, and more specifically in association with planned ocular or corneal surgical interventions.

#### 4.2.2 Corneal complications

Corneal abnormalities are a common finding in patients with congenital aniridia, in view of the progressive AAK which in the most advanced stage results in total corneal opacification and corneal neovascularization (Latta et al., 2021). Although rare, sclerocornea can appear at birth (Alharbi et al., 2021).

Surgical treatment of corneal abnormalities presents a high risk for failure in view of the underlying chronic inflammation, neovascularization and LSCD present in AAK (van Velthoven et al., 2023).

Management of LSCD with limbal stem cell transplantation (LSCT), in the form of keratolimbal allograft (KLAL) transplantation and living‐related conjunctival‐limbal allograft (lr‐CLAL) transplantation, is reported in the literature to have a success rate at 5 years ranging from 25% to 81.4%. (Jacobson et al., 2022; Movahedan et al., 2017; Yazdanpanah et al., 2020). This wide range of outcomes may be related to the different stages of AAK in which LSCT has been performed (Yazdanpanah et al., 2020) and the varying definitions of clinical success, and importantly, there is a lack of controlled studies. Furthermore, LSCT in patients with congenital aniridia cannot be autologous, in view of the bilaterality of the disease, and requires permanent systemic immunosuppression to prevent rejection of the allogeneic tissue (Yazdanpanah et al., 2020). Even newer techniques such as HLA‐matched cultivated limbal epithelial transplantation have resulted in generally poor outcomes in congenital aniridia (Behaegel et al., 2022).

Similar to LSCT, with lack of subgroup comparison for AAK stages, management of corneal opacities with corneal transplantation (with concurrent LSCT or following it) results in a survival rate at 5 years of 43–50%, while with the Boston keratoprosthesis (KPro), the 3‐ to 5‐year retention rate is around 80% (Jacobson et al., 2022; Yazdanpanah et al., 2020).

However, the anatomical success of LSCT, PKP and KPro is usually not followed by clinically significant improvement of visual acuity, which is usually modest at best, and this should be carefully weighed against the burden for patients and risk of complications following these procedures, including long‐term systemic immunosuppression (LSCT, PKP, KPro), worsening of the glaucoma (PKP and KPro), retroprosthetic membrane formation, endophthalmitis and extrusion (KPro). (Jacobson et al., 2022; Viberg et al., 2023; Yazdanpanah et al., 2020).

In view of the above, the decision to perform the procedures is not a routine one and should be carefully considered individually in each case (Viberg et al., 2023), and only after informed consultation with clear communication of risks and realistic outcomes to the patients and caregivers.

### 4.3 Glaucoma

Glaucoma in patients with congenital aniridia has historically been considered difficult to treat, with a poor prognosis (Balekudaru et al., 2017; Landsend et al., 2021).

Considering the prevalence, estimated at 43%–70%, more than half of aniridia patients will develop glaucoma necessitating consistent monitoring of intraocular pressure and appropriate medical and surgical treatment from childhood to adulthood (Landsend et al., 2021).

As mentioned, dysgenesis of the iridocorneal angle and trabecular meshwork is the main cause of glaucoma in aniridia (Grant & Walton, 1974; Swanner et al., 2004), which may be difficult to treat with eye drops, and surgery is often necessary, considering that glaucoma surgery is often challenging to perform (Swanner et al., 2004).

Despite the anterior segment abnormalities, medical treatment is still the first‐line treatment for older children or adolescents. Medical glaucoma treatment must be introduced gradually and maintained for as long as possible to delay the need for surgery. All classes of antiglaucoma topical medication can be used: beta‐blockers, prostaglandin analogues and inhibitors of carbonic anhydrase. Lack of randomized clinical trials and difficulty in conducting trials in view of the rarity of congenital aniridia complicate the recommendation of one class of antiglaucoma medication over another. Among the general population, prostaglandins are the first‐line treatment in adult patients and also have a significant ocular hypotensive effect in children aged 9–13 years, whereas in children younger than 9 years, beta‐blockers are the first‐line treatment (Coppens et al., 2009; Enyedi & Freedman, 2002).

One specific class of antiglaucoma medication, the alpha2‐agonist brimonidine tartrate, is contraindicated under the age of 8 years and should be avoided until the age of 12, as it can have frequent side effects, which include excessive sleepiness and lethargy (76%), eye itching and rubbing (49%), and stinging and burning of the eyes (39%) (Al‐Shahwan et al., 2005). Preservative‐free medications are always recommended if the therapeutic class allows this, as the ocular surface is already compromised in congenital aniridia and preservatives are an additional source of ocular surface toxicity.

In many cases, over time, the intraocular pressure can no longer be adequately controlled with medical treatment and surgical treatment of glaucoma is required. Surgery may consist of: goniotomy (prophylactic and therapeutic), trabeculectomy and glaucoma drainage devices (Landsend et al., 2021). Among these techniques, glaucoma drainage devices appear to have a high success rate (>63%) relative to other techniques, although the literature is very limited concerning congenital aniridia (Jacobson et al., 2022).

In particular, the Baerveldt implant seems more effective, with a success rate (IOP ≤21 mmHg) of 74%, compared to 63% with the Ahmed implant, 24% with trabulectomy combined with anti‐fibrotic medication and 33% with goniotomy (Jacobson et al., 2022).

Regarding prophylactic goniotomy, it must be performed before glaucoma onset and is associated with a risk of damaging the lens and cornea. To date, goniotomy should not be considered as a valid therapeutic approach, in view of the limited number of studies for aniridia (Chen, 1999; Grant & Walton, 1974; Walton, 1986).

Cyclodestructive laser treatments, as cyclocryotherapy and diode laser cyclophotocoagulation, are not indicated and should be considered only as a last resort. Cyclocryotherapy was associated with a low percentage of cases (only 25%) achieving an IOP <21 mmHg, while presenting a higher rate of complications (phthisis and retinal detachment) compared with other cases of paediatric glaucoma (Wiggins, 1992).

Diode laser cyclophotocoagulation was instead associated with a higher frequency of success (50%) (Blake, 1952; Jacobson et al., 2022), but there is a lack of information on follow‐up or complications (Blake, 1952). Additionally, the effect of pressure reduction may only be temporary (Sbordone et al., 2013).

There is a notable lack of studies on the use of non‐destructive laser treatment such as selective laser trabeculoplasty (SLT) in congenital aniridia. For this reason, SLT cannot be considered as a therapeutic option. Similarly, minimally invasive glaucoma surgery and devices, while presenting a promising new approach in the general population, similarly lack evidence in patients with congenital aniridia.

### 4.4 Cataract

As mentioned, congenital cataract is a common finding in patients with congenital aniridia, with an estimated prevalence of up to 90% (Edén et al., 2014; Park et al., 2010). Despite this, cataract surgery should be delayed until lens opacity becomes severe, for two reasons.

The first is that the contribution of cataract in determining the visual acuity in congenital aniridia is significant only in cases of advanced cataract, considering that foveal hypoplasia is the main cause of the severe visual impairment. Therefore, mild to moderate lens opacities may not require surgery. For this reason, children rarely require surgery, whereas adults may experience a greater benefit.

The second reason to delay cataract surgery is the higher risk of complications in aniridia, which include triggering onset of glaucoma or worsening of the existing glaucoma, accelerating the AAK and the risk of triggering corneal endothelial decompensation (Náray et al., 2025; Romano et al., 2023).

Whether these complications are related to cataract surgery and/or simultaneous implantation of artificial iris, however, is unknown, as there is a lack of comparative studies.

Considering the procedure, cataract surgery is more challenging in congenital aniridia, which usually presents with increased zonular weaknesses and a thinner (<8 μm) and more fragile anterior capsule with not only subsequent increased risk of rhexis run‐out but also of anterior capsular tears which may occur during lens manipulation (Neuhann & Neuhann, 2010; Schneider et al., 2003).

In view of the above, capsulorhexis should be performed using capsular staining and cohesive ophthalmic viscosurgical devices. Regarding the size of the rhexis, a more fragile capsule may require a smaller diameter in order to reduce the risk of rhexis run‐out; however, two factors have to be considered (Neuhann & Neuhann, 2010). The first is the pro‐fibrotic status in patients with congenital aniridia, with a subsequent higher risk of triggering fibrosis if anterior capsular phymosis and interference with the intraocular lens optic occur (Neuhann & Neuhann, 2010). The second factor is the concomitant possibility to inject a custom flexible prosthetic iris device in the capsular bag, as a larger rhexis would make the implantation easier (Amaral & Snyder, 2022; Neuhann & Neuhann, 2010).

## 5 PARAMEDICAL MANAGEMENT OF VISUAL IMPAIRMENT

Patients with congenital aniridia benefit from comprehensive care, which includes the active role of non‐medical professionals and an active role for patients.

The first step is to ensure that patients (and in case of children, parents or family members) are fully aware of their condition and how it can impact their daily lives and quality of life.

Patient education should first focus on instructing on the use of rehabilitation tools for visual impairment. This includes training and assessing the patient's knowledge in cases of adults and adolescents, and for young children, training and educating the family. Patient education is also necessary to communicate the potential complications of aniridia, such as ptosis, nystagmus, photophobia, glaucoma, cataract, foveal aplasia, optic nerve or macular hypoplasia and ectopia lentis. Good doctor–patient communication is also important when considering treatment options that include potentially risky interventions that have partial or limited chances of success.

Early education and rehabilitation for vision problems are managed by a specialist team. Patient education measures require coordination between various healthcare professionals and patient associations, who may work with the patient on an individual basis or through group education. A coordinated approach by the various professionals is preferable to a succession of isolated actions.

The visual impairment and photophobia prevalent in congenital aniridia result in visual disability, leading to difficulties with mobility/travel, communication, learning, fine motor skills and independence, with consequences for personal, school, professional, social, cultural and sporting life activities. It should also be kept in mind that *PAX6* aniridia can be linked with developmental and other abnormalities outside the eye that can additionally considerably impact quality of life.

Orthoptists can cooperate with ophthalmologists to assess the visual acuity and help patients learn how to use their residual visual capacity and compensate and/or cope with their visual disability.

This can be achieved, for example using low vision aids, which are beneficial for both children and adults. Optical aids for near vision should aim to enable reading of N4 or N2 prints, by using, for example hand‐held magnifying glasses, microscopes or telescope systems, video magnifiers or computer software for enlarging text. Non‐optical aids include large‐print books with large characters, reading stands, inclined tables, desk lamps with fluorescent tubes (possibly avoiding LED bulbs), writing guides, black felt‐tip pens, tinted corrective lenses (full‐field or reduced‐field, darker on the outside and lighter on the inside).

Optical aids for distance vision can occasionally be used for static purposes, for example reading a street name using a monocular 6× to 8× telescope.

In case of outdoor activities, photochromatic or class 4 corrective lenses are highly recommended for UVA and UVB light protection. Children with aniridia adapt easily to wearing these types of lenses and can attend school wearing just one pair of glasses, which is simple for parents and teachers. Refractive errors must be detected and followed up because adults with aniridia may come to the ophthalmic follow‐up visit without corrective lenses.

Orientation and mobility instructors teach patients how to make the best use of residual visual acuity, advising on how to better understand and navigate in their daily environments to safely move in indoor and outdoor settings. Rehabilitation therapists are additional professionals who guide patients to engage in the activities of daily living and aim to enable patients to gain individual, social and professional independence. (Bremond‐Gignac et al., 2022).

## 6 CURRENT CLINICAL TRIALS

Several clinical trials have been conducted or are ongoing to investigate potential treatments for aniridia and its associated complications. The NCT02647359 trial is a Phase II, multicentre, randomized, double‐masked, placebo‐controlled study evaluating the safety and efficacy of ataluren in patients with nonsense‐mutation‐mediated aniridia. A formulation of ataluren eyedrops has been developed and tested positively (Djayet et al., 2020). An open‐label extension of this study is also registered under NCT04117880, focusing on long‐term systemic and ocular safety. Another significant study is NCT05044598, a first‐in‐human Phase I/II trial exploring the use of RAFT‐OS, a novel tissue‐engineered product using limbal epithelial and stromal cells, aimed at treating advanced AAK. These trials collectively reflect ongoing efforts to improve management options for aniridia through both pharmacologic and regenerative approaches.

## 7 CONCLUSION

Congenital aniridia is a complex panocular disorder affecting the majority of the eye's structures and can also have systemic manifestations. Because of this complexity, the management of patients is challenging, requiring a multidisciplinary approach with both medical and non‐medical professionals collaborating.

Considering the known outcomes of treatments for the ocular comorbidities in congenital aniridia, in the case of glaucoma, a precise goal exists, whereas for other complex procedures (PID, KPro, LSCT and corneal transplantation), the improvement of visual acuity should not be considered as the main goal and the choice of further procedures should be based on symptom‐related difficulties experienced by patients.

Given that in congenital aniridia, the main cause of visual impairment is foveal hypoplasia, and in later stages, AAK, the improvement of visual acuity following many procedures is usually modest and the risk/benefit must be carefully weighed. It may be more beneficial to focus on the improvement of symptoms and health‐related quality of life, possibly measured with standardized questionnaires, rather than focusing solely on improving visual acuity as a goal (Hoxha et al., 2025; Landsend et al., 2024).

Another aspect to be considered is that the scientific literature concerning congenital aniridia currently lacks randomized controlled studies providing a high level of evidence. Such evidence, however, would be challenging to obtain on ethical grounds where a potentially beneficial treatment would need to be withheld. Additionally, a highly variable and heterogeneous presentation of congenital aniridia patients makes comparative studies difficult, also given the presence of multiple ocular comorbidities.

Our aim with these guidelines was to provide an updated overview on current clinical practice and recommendations concerning the management of patients with congenital aniridia, an update on current treatment options and their outcomes and how to set up a comprehensive plan of care and management by medical and non‐medical professionals.
